# Casting Light on The Hidden Prevalence: A Novel Perspective on Hypoplastic Coronary Artery Disease

**DOI:** 10.3390/jcm13092555

**Published:** 2024-04-26

**Authors:** Alexandra-Simona Zamfir, Cristian Stătescu, Radu Andy Sascău, Grigore Tinică, Carmen Lăcrămioara Zamfir, Tudor-Andrei Cernomaz, Raluca Ozana Chistol, Daniela Boișteanu, Anca Sava

**Affiliations:** 1 Clinical Hospital of Pulmonary Diseases, 700115 Iași, Romania; 2 Department of Medical Sciences III, Faculty of Medicine, “Grigore T. Popa” University of Medicine and Pharmacy, 700115 Iași, Romania; 3 Department of Medical Sciences I, Faculty of Medicine, “Grigore T. Popa” University of Medicine and Pharmacy, 700115 Iași, Romania; 4 Cardiology Department, “Prof. Dr. George I.M. Georgescu” Cardiovascular Diseases Institute, 700503 Iași, Romania; 5 Department of Surgery I, Faculty of Medicine, “Grigore T. Popa” University of Medicine and Pharmacy, 700115 Iași, Romania; 6 Department of Cardiovascular Surgery, “Prof. Dr. George I.M. Georgescu” Cardiovascular Diseases Institute, 700503 Iași, Romania; 7 Department of Morpho-Functional Sciences I, Faculty of Medicine, “Grigore T. Popa” University of Medicine and Pharmacy, 700115 Iași, Romania; 8 Regional Institute of Oncology, 700483 Iași, Romania; 9 Department of Medical Imaging, “Prof. Dr. George I.M. Georgescu” Cardiovascular Diseases Institute, 700503 Iași, Romania; 10 Department of Pathology, “Prof. Dr. Nicolae Oblu” Emergency Clinical Hospital, 700309 Iaşi, Romania

**Keywords:** cardiovascular congenital disorders, coronary artery anomalies, hypoplastic coronary arteries, coronary computed tomography angiography (CCTA)

## Abstract

**Background and Objectives:** Coronary artery anomalies (CAAs) represent a group of rare cardiac abnormalities with an incidence of up to 1.2%. The aim of this retrospective study was to conduct a comprehensive epidemiological assessment of the prevalence of hypoplastic coronary arteries using coronary computed tomography angiography (CCTA) in patients with diagnosed CAAs and individuals presenting with cardiovascular manifestations in the north-eastern region of Romania. This study was motivated by the limited investigation of the CAAs conducted in this area. **Methods:** We analyzed data collected from 12,758 coronary computed tomography angiography (CCTA) records available at the “Prof. Dr. George I.M. Georgescu” Cardiovascular Diseases Institute, spanning the years 2012 to 2022. **Results:** Among 350 individuals with CAAs (2.7% of the total cohort), 71 patients (20.3% of the anomaly presenting group and 0.5% of the entire CCTA cohort) exhibited at least one hypoplastic coronary artery. The mean age of individuals diagnosed with hypoplastic coronary artery disease (HCAD) was 61 years, while the age distribution among them ranged from 22 to 84 years. Nearly equal cases of right and left dominance (33 and 31, respectively) were observed, with only 7 cases of co-dominance. **Conclusions:** HCAD may be considered underexplored in current published research, despite its potentially significant implications ranging to an increased risk of sudden cardiac arrest. The specific prevalence of HCAD among CAAs might be higher than previously reported, possibly reflecting better diagnostic accuracy of CCTA over classic coronary imaging. The absence of standard diagnostic and therapeutic protocols for HCAD underscores the necessity of a personalized approach for such cases.

## 1. Introduction

### 1.1. Background

The coronary artery anomalies (CAAs) represent an umbrella term encompassing cardiovascular congenital disorders, spanning a wide spectrum from asymptomatic cases to severe and even potentially malignant forms [1]. The incidence of different CAAs is low and exhibits variability across diverse geographical regions [2,3]. The variability in detecting coronary artery anomalies is largely influenced by disparities in investigative approaches. Post-mortem examinations are commonly conducted in cases of sudden cardiac death among younger individuals, while patients with suspected or known CAAs are frequently directed towards specialized angiographic centers for invasive procedures. For example, in the most extensive angiographic study to date, involving 126,595 patients, Yamanaka and Hobbs documented a prevalence of anomalous coronary arteries at 1.2% [3]. On the other hand, the widespread adoption of non-invasive coronary computed tomography angiography (CCTA) has provided additional insights into the epidemiological landscape of CAAs, revealing a potentially higher prevalence. CCTA provides comprehensive insights into high-risk CAAs, offers in-depth imaging of both cardiac and extracardiac structures, and facilitates population studies due to its broader applicability [1]. Furthermore the better safety profile of CCTA makes it more likely to be requested and performed; expanding the population scanned might change the detected prevalence of various coronary anomalies. 

Although initially noted in the 18th century, their relevance and classification were formally established in 1969, with subsequent revisions in 2000 [1]. In terms of their functional relevance, coronary artery anomalies often present a captivating paradox. While some anomalies may exist quietly, devoid of symptoms, and only come to light incidentally, others can wield a substantial impact, ushering in severe clinical manifestations and, in some cases, precipitating ischemic events. This duality underscores the complexity of CAAs and the diverse array of clinical scenarios they can present, provoking ongoing debate and inquiry within the medical area. Furthermore, it’s noteworthy that the same anomaly can manifest differently in each individual, adding another layer of complexity to their clinical significance and management [4]. 

Angelini’s classification system stands as one of the most extensive frameworks for categorizing coronary artery anomalies (CAAs), offering a comprehensive approach to understanding their diverse anatomical aspects [5]. 

Within this classification, a hypoplastic coronary artery is designated as a congenital intrinsic anatomical anomaly. This anomaly manifests when one or more of the epicardial coronary arteries, including the right coronary artery, left coronary artery, left anterior descending artery, or left circumflex artery, undergo restricted growth. This reduction in size is characterized by a significant decrease in either the diameter or length of the affected artery [6]. A threshold value of 1.5 mm is commonly employed as a diagnostic criterion to identify hypoplastic coronary arteries [7]. This classification underscores the importance of recognizing and categorizing hypoplastic coronary arteries within the spectrum of CAAs, facilitating management strategies in clinical practice. 

Initially described in 1970, the clinical expression of this congenital disorder is exemplified by the hypoplastic coronary artery disease (HCAD). This disorder encompasses a spectrum of manifestations ranging from mild symptoms such as chest pain or dyspnea to more severe outcomes, including syncope or sudden death. Among various other coronary abnormalities, the prevalence of HCAD remains elusive, with fewer than 30 case reports documented in the literature. In this limited pool of reported cases, the right coronary artery (RCA) and left circumflex artery (LCX) are commonly implicated [8]. Nevertheless, to date, no study has elucidated the mortality rates associated with hypoplastic coronary arteries. Moreover, their clinical significance extends beyond their congenital origins. While they may manifest early in life or remain asymptomatic until adulthood, hypoplastic coronary arteries can pose significant clinical challenges and complications throughout a patient’s lifespan. By acknowledging the interplay between congenital predispositions, acquired factors, and clinical outcomes, clinicians and researchers can endeavor towards a more holistic understanding of HCAD. 

To date, existing literature comprises mainly isolated case reports correlating hypoplastic coronary arteries with instances of sudden death or individual cases, rather than offering a comprehensive review of this pathological condition. Debates surround the challenges in effectively identifying and managing hypoplastic coronary arteries due to their subtle presentation and limited data availability, potentially leading to delays in diagnosis and underestimation of prevalence. Moreover, historical limitations in diagnostic techniques may have hindered the accurate identification of hypoplastic coronary arteries in the past, emphasizing the importance of advancing diagnostic modalities and refining clinical practices to enhance detection rates.

### 1.2. Aims

We aimed to estimate the prevalence of hypoplastic coronary arteries in the north east region of Romania using a retrospective approach on existing CCTA data available in the medical repository of “Prof. Dr. George I.M. Georgescu” Cardiovascular Diseases Institute, Iasi, Romania. Since no CCTA data is available on a general population basis, we used a surrogate population consisting of patients with cardiovascular signs and symptoms referred to our tertiary unit.

## 2. Materials and Methods

### 2.1. Study Design and Patients

A total of 12,758 coronary computed tomography angiography (CCTA) scans obtained between 2012 and 2022 were considered. The CCTAs were performed on patients with known cardiovascular conditions or referred for clinical suspicions of ischemic heart disease according to local protocols. 

Data pertaining to the putative presence of hypoplastic coronary arteries was meticulously recorded and analyzed utilizing established diagnostic criteria; detailed radiological assessments were performed by experienced radiologists trained in cardiovascular imaging. Hypoplasia in coronary arteries was defined by a notable reduction in calibre or extent. A commonly accepted diagnostic threshold of 1.5 mm was utilized to discern significant diminishment indicative of hypoplastic coronary arteries. Additionally, information about the dominance of the coronary arteries was noted. The study design and protocol were approved by the Ethics Committee of “Grigore T. Popa” University of Medicine and Pharmacy Iasi, Romania (no. 115/15.10.2021).

**Figure 1:**
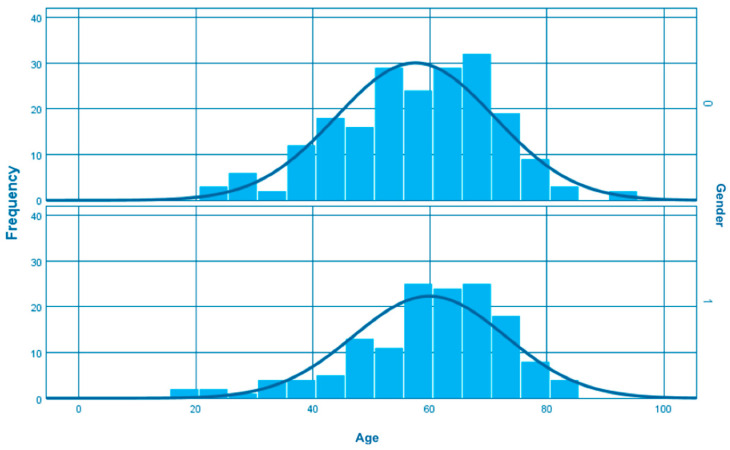
Age distribution among patients with coronary artery anomalies (0–men, 1–female).

A total of 350 patients with coronary artery anomalies were identified in our dataset. Among these individuals, 204 (58.3%) were male and 146 (41.7%) were female. The median age was 58.49 years showing slight differences between genders, as men presented a median age of 57.5 and women 59.9 years (Figure 1).

As the study group is not a reliable sample of the general population, we considered useful to assess the presence of various cardiovascular risk factors and other potentially clinical significant conditions—as this data might help framing and interpreting the results. We evaluated the primary cardiovascular risk factors including hypertension (blood pressure values ≥140/90 mmHg), diabetes (hemoglobin A1C ≥6.5%), dyslipidemia (total cholesterol >5.2 mmol/L (200 mg/dL), LDL > 3.4 mmol/L (130 mg/dL), HDL <0.9 mmol/L (35 mg/dL), or triglycerides >1.7 mmol/L (150 mg/dL)), and smoking status, specifically within the cohort of patients diagnosed with coronary artery anomalies (Table 1). 

Furthermore, we documented the presence of coronary artery disease (CAD) or chronic cardiac failure in our study cohort (Table 2).

At the same time, we recorded any therapeutic interventions undertaken, including coronary angioplasty, pacemaker insertion, and surgical bypass procedures (Table 3).

### 2.2. Statistical Analysis

The statistical analysis was conducted using Statistical Package for Social Sciences v.27 (IBM Corp., Armonk, NY, USA). Normally distributed continuous variables are presented as average ± standard deviation; categorical variables are displayed as absolute frequencies and percentages relative to the total number of sub-groups.

## 3. Results

Within the studied cohort encompassing 12,758 patients, 350 individuals, constituting 2.7% of the sample, exhibited a coronary artery anomaly—the most prevalent are the absent left main, coronary artery hypoplasia and myocardial bridges (Table 4).

**Figure 2:**
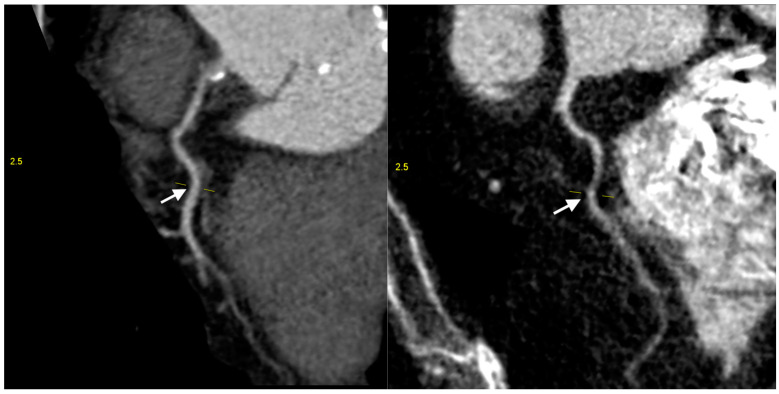
Coronary computed tomography angiography of hypoplastic right coronary arteries (white arrow) (images are upscaled to a 2.5 factor).

Among these anomaly-affected individuals, 71 patients, accounting for 20.3% of the anomaly-presenting group, showcased at least one hypoplastic coronary artery (Figure 2). 

**Figure 3:**
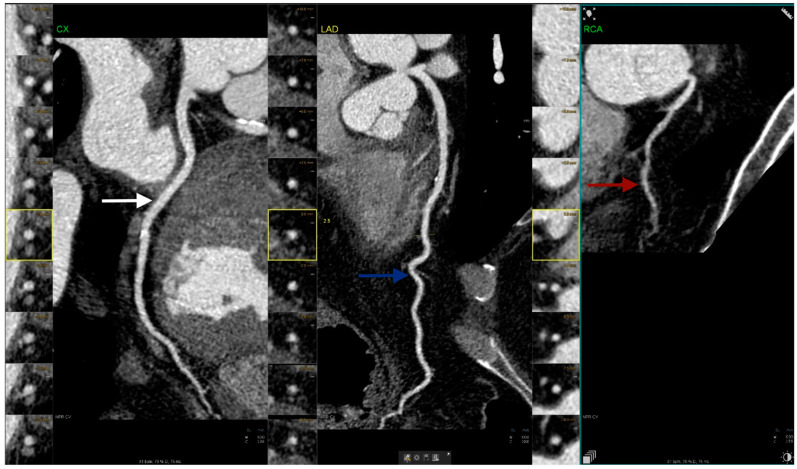
Coronary computed tomography angiography figure illustrates a case of right hypoplastic coronary artery (red arrow) alongside normal left circumflex artery (white arrow) and left anterior descending artery (blue arrow) (images are upscaled to a 2.5 factor).

Notably, preceded only by the absence of the left main trunk (split left coronary artery), hypoplasia emerges as the second most prevalent anomaly in our analysis, closely aligning with the primary focus of our article (Figure 3). 

**Figure 4:**
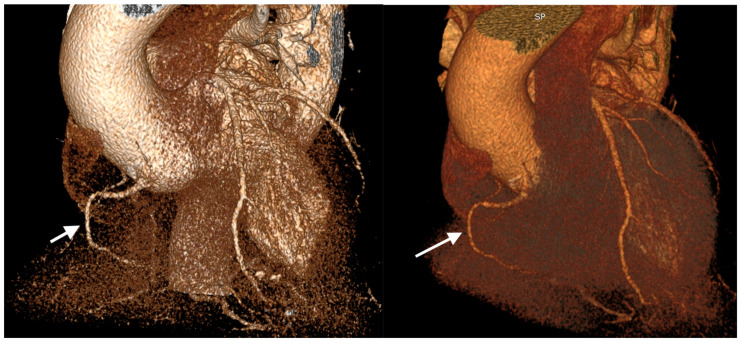
3-dimensional volume-rendered images obtained from coronary computed tomography angiography in a 60-year-old male patient with hypoplastic right coronary (white arrow) presented with cardiac failure.

The mean age of individuals diagnosed with HCAD was 61.61 years, while the age distribution among them ranged from 22 to 84 years. Within the identified patients, there was a modest variation in gender distribution, with 39 cases of coronary hypoplasia recorded in males (54.9%) and 32 in females (45.1%) (Figure 4).

In examining the coronary dominance, we observed a nearly equivalent representation of right and left dominance (33 and 31 cases, respectively), while only 7 patients presented co-dominance.

In assessing the cardiovascular risk factors observed among these patients, it is evident that a considerable proportion exhibited hypertension, with 33 individuals (47%) affected. Dyslipidemia was present in 23 cases (32%), while diabetes mellitus and smoking each accounted for 8 cases (11%) (Table 5).

A total of 26 patients (36.6%), evenly split between genders with 13 men and 13 women, presented with chronic cardiac failure. Additionally, 24 individuals (33.8%), comprising 19 men and 5 women, exhibited coronary artery disease.

In our research, we observed stent placement in 10% of the patients from the study, comprising six men and one woman, making this intervention more frequent in men with HCAD (Table 6). Among the seven cases identified, three involved a hypoplastic left main coronary artery (LM), with stents placed in its branches: two in the left anterior descending artery (LAD) and one in the left circumflex artery (LCx). Notably, in one case, the hypoplastic artery was the LAD itself, and a stent was placed in this artery. This represented the sole instance of stent placement directly on the hypoplastic artery. In the remaining cases, despite the presence of a hypoplastic right coronary artery, a stent was placed in the LCx. Conversely, in another patient, despite a hypoplastic LCx, stenosis was observed in the RCA.

At the same time, 2 female patients (2.8%) presented pacemaker implantation. One was a 56-year-old woman with right coronary artery hypoplasia who associated complete heart block, while the second involved a 49-year-old woman presenting left main hypoplasia and ventricular tachycardia.

Concerning other therapeutic interventions among patients with HCAD, we identified two instances of bypass surgery among male individuals with right coronary hypoplasia (2.8%). The first patient underwent quadruple coronary artery bypass grafting (CABG), involving two permeable grafts. This procedure utilized the left internal mammary artery (LIMA) to bypass the left anterior descending artery (LAD), and a saphenous vein graft (SVG) to bypass the left posterolateral artery (LPL). In the second case, a male patient underwent triple coronary artery bypass grafting, utilizing the left internal mammary artery for the left anterior descending artery, and segments of the internal saphenous vein for the diagonal and obtuse marginal arteries.

## 4. Discussion

Coronary artery anomalies represent a relatively uncommon spectrum of conditions, exhibiting a diverse range of clinical presentations that may vary from benign to malignant. These anomalies generally exhibit a prevalence ranging from 0.17% in postmortem examinations to 1.2% in cases evaluated via angiography [3]. This variability underscores the challenges in accurately assessing the true incidence of coronary artery anomalies, as their detection heavily relies on diagnostic methodologies and the population under study. Additionally, the discrepancy between autopsy and angiographic findings highlights the potential underestimation of these anomalies in clinical practice, warranting further investigation and awareness to ensure comprehensive detection. Currently, there is a growing emphasis on the non-invasive detection of CAAs. Within this framework, CCTA has emerged as a preferred modality over invasive angiography. Notably, CCTA exhibits superior accuracy in identifying CAAs [9]. While cardiovascular magnetic resonance imaging (CMR) may circumvent this issue, it is associated with higher costs and relatively limited availability compared to CCTA. Moreover, it correlates with significantly longer scanning procedures and may exhibit inferior accuracy compared to CCTA [10]. CCTA demonstrates superior performance in detecting CAAs compared to CMR, with a notably lower diagnostic failure rate of 9.6% for CCTA versus 59.6% for CMR [11]. 

There are some evident limitations of the presented analysis—partially derived from the retrospective approach and also from the nature of the exploratory intervention. While CCTA is non-invasive and has a better safety profile than classic coronary angiography, it still carries the risks and limitations associated with ionizing radiation exposure. Therefore, it is difficult or unacceptable from a risk/benefit point of view to conduct real-world studies on the prevalence of coronary artery anomalies in the general population. However, there is still data available concerning population subgroups—such as patients presenting cardiovascular symptoms. Such an approach is not new—previous data on coronary variants and abnormalities were obtained from similar populations, sometimes postmortem, which may add significant bias [12]. From this point of view, assessing the prevalence using the CCTA data has the potential to be closer to the real values.

At the same time, despite the strong correlation between body size and coronary artery diameter, an intriguing phenomenon arises: women consistently exhibit smaller coronary arteries than men, even after accounting for differences in body size [13]. However, it’s worth noting that in our study, the majority of patients exhibiting hypoplasia of the coronary arteries were men.

While angiographic studies suggest a correlation between coronary artery diameter and height, implying a potential link between stature and CAD, it’s essential to consider the broader context. This intricate relationship between anthropometric measures and cardiovascular health isn’t solely about height; weight also plays a crucial role. Factors like perinatal nutrition, exposure to environmental toxins such as tobacco, socioeconomic disparities, and genetic predispositions may contribute to vascular damage [14].

Angelini’s classification system delineates CAAs into four distinct categories based on their anatomical characteristics: anomalies of origination and course, anomalies of intrinsic coronary anatomy, anomalies of coronary termination and anomalous collateral vessels. Hypoplasia, the specific anomaly under investigation in our study, falls within the category of intrinsic anatomical anomalies [5].

Hypoplastic coronary artery disease (HCAD) represents a congenital anomaly characterized by the inadequate development or diminutive size of at least one of the principal coronary arteries or one of its branches, often accompanied by luminal constriction or abbreviated course [8]. This description underscores the pathological significance of HCAD in contributing to coronary insufficiency and potential cardiovascular sequelae. Its initial documentation dates back to 1970 [8]. However, although recognized over five decades ago, HCAD remains relatively underexplored in the medical literature.

Despite its potential clinical involvement, a notable omission of consideration of HCAD persists in numerous studies and clinical discussions, regardless of the study type (post-mortem, angiographic or CCTA investigations). Ogden conducted a study on 224 hearts with coronary artery anomalies, revealing a 2.2% incidence of hypoplastic coronary arteries among the patient cohort examined through postmortem examination [12]. This study stands as the most relevant to date for investigating the prevalence of hypoplastic coronary incidence among coronary artery anomalies. Another research conducted in 2018, involving only 50 cadaveric hearts, reveals that 6 of the hearts presented hypoplastic left coronary artery (LCA), while two had hypoplastic LCx, encompassing 12% of the sample [15]. Contrarily, in our study, employing CCTA, the specific incidence of HCAD among patients with CAAs was observed to be higher at 20.3%. This substantial difference prompts a critical inquiry into the potential underdiagnosis of HCAD within clinical practice. The difference may potentially be explained by selection bias present in both our study group and the original 1970 Ogden’s work, as there is not a standard screening or diagnosis protocol to identify CAAs in asymptomatic patients, neither then nor presently [12]. Coronary artery anatomy assessment remains a standard procedure for patients exhibiting a constellation of clinical manifestations and risk factors, with some variability owing to local protocols or availability of medical procedures. Another source of bias may be the method of assessment—post-mortem procedures versus coronary angiography versus CCTA [9,16]. There is data supporting better detection when using CCTA compared to other methods in detecting not only hypoplasia, but also other arterial anomalies. Nowadays, with the development of CCTA, the potential for their detection has increased [9]. 

Our research emphasized the utilization of CCTA owing to its high diagnostic accuracy in detecting CAAs. Presently, no standardized screening protocol exists for identifying CAAs in asymptomatic individuals. Although specific clinical presentations, such as syncope or severe arrhythmias in young patients, may prompt consideration for CCTA, it is pertinent to acknowledge that asymptomatic individuals without known cardiac conditions typically do not undergo this imaging modality. Consequently, our study specifically targeted patients admitted to the hospital due to coronary artery disease and other cardiovascular manifestations, reflecting a population wherein CCTA is integral as part of their clinical assessment.

Nitric oxyide synthase (NOS3) plays an important role in fostering the development of the arterial coronary system. It accomplishes this function by enhancing the expression of transcription and growth factors and facilitating the movement of the cells derived from the epicardium into the myocardium. Recent data support the fact that the deficiency of NOS3 contributes to the development of hypoplastic coronary arteries and constitutes an initial stride towards unravelling the molecular mechanism underlying HCAD, indicating its potential as a valuable model for further investigations [17,18]. Previously, research focused on delineating how pregestational diabetes detrimentally influences the epicardial epithelial-to-mesenchymal transition (EMT), leading to a reduction in coronary arteries volume in mice. Oral administration of N-acetylcysteine to pregnant women with diabetes appears to prevent or reduce the congenital heart malformations. These findings hint at a therapeutic avenue for mitigating congenital heart defects, including coronary artery malformation, in offspring born to mothers with pregestational diabetes [19]. Another hypothesis is concentrated on the involvement of maternal nicotine exposure (MNE) in the development of hypoplastic coronary arteries. Recent studies on mice demonstrated that fetal exposure to MNE at a dosage of 1.5 mg/kg/day is associated with decreased diameters of both left and right coronary arteries. At the same time, MNE at dosages of 0.75 and 1.5 mg/kg/day led to the induction of coronary artery malformations in 4% and 30% of the fetuses [20].

Angelini stated that this anomaly, correlated with diminished maximal flow reserve alongside normal resting flow, may constitute an underlying substrate involved in some patients with X Syndrome, represented by myocardial ischemia lacking significant coronary stenosis. A similar discordance has been hypothesized as a mechanistic basis for cardiomyopathy, observed in experimental models of chronic ventricular overload [21]. At the same time, another study elaborated by Maron et al. delved into cases of sudden death among athletes, and concluded that among the 48 subjects diagnosed with cardiomyopathy, six had CAAs and two of them presented HCAD [22]. Additionally, two isolated cases of a 17-year-old girl and a 30-year-old man who suffered of sudden death associated with sport events were demonstrated to present both hypoplasia of the right coronary artery (RCA) and left circumflex artery (LCx) [4]. This matter underscores the intricate pathophysiological mechanism contributing to certain cardiovascular disorders, especially those which involve ischemia without any overt coronary artery obstruction. While intriguing, this hypothesis needs further rigorous studies to validate its clinical significance.

As previously stated, in severe instances, the primary clinical manifestation of HCAD is usually represented by sudden death, rendering this anomaly predominantly detectable during autopsies. The majority of the documented cases have involved people engaged in competitive sports. Less frequently, the initial presentation in these patients includes myocardial infarction, precipitating arrhythmias [23,24]. It is noteworthy to highlight that the underlying mechanisms governing myocardial ischemia in the context of CAAs remain a challenge for clinicians and researchers alike. Nevertheless, alternative manifestations of HCAD encompass angina, syncope, dyspnea or irregular heartbeats [24].

During the analysis of the age of the identified patients, an interesting observation emerges regarding the diverse age distribution among those identified. While their identification in younger patients may signify early manifestations of the anomalies or concomitant cardiac conditions, in older individuals their presence may be correlated with age-related vascular alteration, suggesting a complex interplay between age and anomaly presentation. At the same time, the observed mean age of 61.61 years within the context of HCAD can be understood, particularly when considering the reported incidence of cardiovascular disease, which stands at 77.2% in men and 78.2% in women aged 60–79 years [25]. At the same time, our study highlights a subtle, but noteworthy difference in the prevalence of coronary hypoplasia between genders. While the slightly predominance of HCAD in men may not be substantial, it underscores the importance of exploring factors (such as genetic predispositions or acquired factors which disturb the coronary flow) that may contribute to the manifestation of coronary hypoplasia differently between men and women.

Despite approximately 85% of the general population being considered to have a right coronary dominance, wherein the RCA is responsible to irrigate the cardiac inferior wall and also supplies the right posterior descending artery (PDA), it is noteworthy that in the context of HCAD, there exists an almost equal distribution between the right (46.5%) and left coronary dominance (43.7%) [26]. Left dominance, characterized by the left circumflex artery (LCx) providing the PDA, is considered to have a reduced prevalence with advanced age. This observation may be attributed to the increased myocardial territory at risk inherent in anterolateral myocardial infarctions occurring within these individuals [26,27]. These patterns suggest potential hemodynamic adaptations and compensatory mechanism at play, considering the compromised size or function of the affected coronary artery. Consequently, the adjustments in blood flow distribution may lead to a more balanced dominance pattern between the right and left coronary arteries. Simultaneously, co-dominance situations, wherein both RCA and LCx supply PDA, are encountered in less than 10% of the patients with HCAD, a proportion nearly similar to the one of the general population (7–8%) [26]. 

Regarding the involved cardiovascular risk, hypertension was evident in nearly half of the studied cases. Prior investigations failed to establish a concrete correlation between the presence of HCAD and the development of hypertension. Nevertheless, among 17 documented pediatric cases, two exhibited hypertension, posited as a compensatory mechanism at preserving coronary perfusion [28]. At the same time, it is important to remember that hypertension stands as a primary precipitant in the development of CAD. In the case of patients with HCAD, it may further impact the hemodynamic stress, potentially exacerbating associated complications such as myocardial ischemia [29]. Simultaneously, dyslipidemia and diabetes mellitus increase the risk of CAD, owing to their involvement in endothelial and microvascular dysfunction [30]. Additionally, smoking was identified in just 8 individuals, and its established association with heightened risk of acute myocardial infarction suggests a potential increased impact on the already compromised coronary circulation in the affected patients [31].

Different hypothesis have been developed regarding the therapeutic options available for patients with HCAD. Several studies suggest that an implantable cardioverter-defibrillator (ICD) may be optimized as a treatment modality for the patients experiencing severe arrhythmia or those deemed at increased risk of sudden cardiac death. Despite the absence of recommendations or descriptions of bypass surgery as a solution in the previously reported cases, heart transplantation emerges as a viable consideration in the instances with severe or end stage cardiac failure [24,32].

In our investigation, a low frequency of bypass surgeries and pacemaker implantations was noted, underscoring the demand for personalized management strategies for patients. Despite not being directly associated with the CAAs, the occurrence of these interventions within this context prompts further exploration into their potential implications for patient care and underscores the complexity of managing such cardiac conditions. At the same time, nearly 10% of the patients required stent placement. An intriguing observation arises from the fact that only one patient was female, while 6 were males. 

The observed patterns of stent placement in patients with hypoplastic coronary arteries raise intriguing questions about the interplay between atherosclerosis, coronary anatomy, and clinical management strategies. In cases where the hypoplastic artery is the left main coronary artery (LM), and stents are deployed in its branches such as the left anterior descending artery (LAD) or left circumflex artery (LCx), the association with atherosclerosis may be multifaceted. While LM hypoplasia itself may not directly contribute to atherosclerosis, alterations in coronary flow dynamics resulting from the hypoplastic anatomy could potentially influence the development and distribution of atherosclerotic plaque within the coronary circulation. Moreover, the need for stent placement in the LAD or LCx branches of a hypoplastic LM may reflect the presence of significant atherosclerotic lesions, necessitating intervention to maintain myocardial perfusion. Conversely, the singular case where the hypoplastic artery was the LAD, and stent placement occurred in the same vessel, highlights an interesting scenario where stenting was directly targeted towards the hypoplastic segment. While this situation may suggest a direct association between hypoplasia and atherosclerosis, further investigation is warranted to elucidate the underlying mechanisms and clinical implications. In the remaining cases where the hypoplastic artery were the RCA or LCx and stents were placed in other coronary arteries, the relationship between hypoplasia and atherosclerosis may be less straightforward. Here, the decision to perform stent placement in non-hypoplastic arteries could be driven primarily by the severity and location of atherosclerotic lesions rather than the hypoplastic nature of the coronary arteries. However, the presence of hypoplastic coronary arteries may still influence coronary hemodynamics and plaque distribution, potentially impacting the overall clinical presentation and management of atherosclerotic disease in these patients.

Taking all these results into consideration, it is pertinent to underscore that there is no standardized treatment protocol for patients diagnosed with HCAD. Instead, an individualised therapeutic approach is warranted to effectively address the underlying pathology and associated complications, with the ultimate goal of optimizing outcomes and enhancing both quality of life and life expectancy. 

## 5. Conclusions

Hypoplastic coronary artery disease (HCAD) may be considered underexplored in current published research, despite its potentially significant implications ranging to an increased risk of sudden cardiac arrest. The specific prevalence of HCAD among coronary artery anomalies might be higher than previously reported, possibly reflecting better diagnostic accuracy of CCTA over classic coronary imaging. The absence of standard diagnostic and therapeutic protocols for HCAD underscores the necessity of a personalized approach for such cases.

## Figures and Tables

**Table 1:** Prevalence of cardiovascular risk factors in patients with CAAs.

Risk Factors	Total (*n* = 350)		Men (*n* = 204)		Female (*n* = 146)	
	*n*	%	*n*	%	*n*	%
Hypertension	149	42.6%	95	46.5%	54	37%
Dyslipidemia	97	27.7%	54	12.7%	43	12.3%
Smoking	45	12.9%	32	15.6%	13	9%
Diabetes mellitus	44	12.6%	26	12.7%	18	12.3%

Values truncated for brevity.

**Table 2:** Prevalence of coronary artery disease and chronic cardiac failure in patients with CAAs.

Cardiovascular Diseases	Total (*n* = 350)		Men (*n* = 204)		Female (*n* = 146)	
	*n*	%	*n*	%	*n*	%
Coronary artery disease (CAD)	108	30.9%	77	37.7%	31	21.2%
Chronic cardiac failure	121	34.6%	69	33.8%	52	35.6%

Values truncated for brevity.

**Table 3:** Therapeutic interventions in patients with CAAs.

Cardiovascular Diseases	Total (*n* = 350)		Men (*n* = 204)		Female (*n* = 146)	
	*n*	%	*n*	%	*n*	%
Coronary angioplasty	29	8.3%	23	11.3%	6	4%
Pacemaker insertion	10	3%	5	2.4%	5	3.4%
Bypass	5	1.4%	5	2.4%	0	0%

Values truncated for brevity.

**Table 4:** Prevalence of the three most frequent coronary artery anomalies identified in the study.

Type of Anomaly	Number of Anomalies	Coronary Artery Anomalies (%) (*n* = 350)	Incidence (%) (*n* = 12,758)
Absent left main trunk (split origination of LCA)	80	22.9%	0.6%
Coronary artery hypoplasia	71	20.3%	0.5%
Myocardial bridges	66	18.9%	0.5%

Values truncated for brevity.

**Table 5:** Prevalence of cardiovascular risk factors in patients with HCAD.

Risk Factors	Total (*n* = 71)		Men (*n* = 39)		Female (*n* = 32)	
	*n*	%	*n*	%	*n*	%
Hypertension	33	47%	20	51%	13	41%
Dyslipidemia	23	32%	13	33%	10	31%
Smoking	8	11%	6	15%	2	6%
Diabetes mellitus	8	11%	5	13%	3	9%

Values truncated for brevity.

**Table 6:** Characteristic of stent placement in patients with HCAD (M-male, F-female, LM-left main, RCA-right coronary artery, LAD-left anterior descending artery, LCx-left circumflex coronary artery).

Patients (*n*)	Gender	Hypoplastic Coronary Artery	Stenotic Coronary Artery with Stent Placement
1	M	LM	LAD
2	M	RCA	LAD
3	F	LAD	LAD
4	M	LM	LAD
5	M	RCA	LCx
6	M	LM	LCx
7	M	LAD	RCA

## Data Availability

Data are contained within the article.

## References

[1] Gentile F., Castiglione V., De Caterina R. (2021). Coronary Artery Anomalies. Circulation.

[2] Jiang X., Zhou P., Wen C., Yin Z., Liu T., Xu M., Yang C., Wang H., Song W., Fang Y. (2021). Coronary Anomalies in 11,267 Southwest Chinese Patients Determined by Angiography. Biomed. Res. Int..

[3] Graidis C., Dimitriadis D., Karasavvidis V., Dimitriadis G., Argyropoulou E., Economou F., George D., Antoniou A., Karakostas G. (2015). Prevalence and characteristics of coronary artery anomalies in an adult population undergoing multidetector-row computed tomography for the evaluation of coronary artery disease. BMC Cardiovasc. Disord..

[4] Villa A.D., Sammut E., Nair A., Rajani R., Bonamini R., Chiribiri A. (2016). Coronary artery anomalies overview: The normal and the abnormal. World J. Radiol..

[5] Kastellanos S., Aznaouridis K., Vlachopoulos C., Tsiamis E., Oikonomou E., Tousoulis D. (2018). Overview of coronary artery variants, aberrations and anomalies. World J. Cardiol..

[6] Yazici H.U., Aydar Y., Birdane A., Ulus T., Nadir A., Kirli I., Gorenek B., Unalir A., Ata N. (2013). Relationship between hypoplastic right coronary artery and coronary artery anomalies. Eur. Rev. Med. Pharmacol. Sci..

[7] Zugibe F.T., Zugibe F.T., Costello J.T., Breithaupt M.K. (1993). Hypoplastic Coronary Artery Disease Within the Spectrum of Sudden Unexpected Death in Young and Middle Age Adults. Am. J. Forensic Med. Pathol..

[8] Guo A., Bakhshi H., O’Hara J., Genovese L., Fein A., Maghsoudi A., Sandesara C. (2021). Hypoplastic Coronary Artery Disease Presenting with Ventricular Fibrillation Cardiac Arrest. Eur. J. Case Rep. Intern. Med..

[9] Ghadri J.R., Kazakauskaite E., Braunschweig S., A Burger I., Frank M., Fiechter M., Gebhard C., A Fuchs T., Templin C., Gaemperli O. (2014). Congenital coronary anomalies detected by coronary computed tomography compared to invasive coronary angiography. BMC Cardiovasc. Disord..

[10] Al Umairi R., Al-khouri M. (2019). Prevalence, Spectrum, and Outcomes of Single Coronary Artery Detected on Coronary Computed Tomography Angiography (CCTA). Radiol. Res. Pract..

[11] van Stijn D., Planken N., Kuipers I., Kuijpers T. (2021). CT Angiography or Cardiac MRI for Detection of Coronary Artery Aneurysms in Kawasaki Disease. Front. Pediatr..

[12] Ogden J.A. (1970). Congenital anomalies of the coronary arteries. Am. J. Cardiol..

[13] O’Connor N.J., Morton J.R., Birkmeyer J.D., Olmstead E.M., O’Connor G.T. (1996). Effect of Coronary Artery Diameter in Patients Undergoing Coronary Bypass Surgery. Circulation.

[14] Yano S. (2022). Does Body Height Affect Vascular Function?. Hypertens. Res. J..

[15] Manickavasuki A.K., Jamuna M., Hebzibah T.D., Nirmaladevi M., Swamicken B.P., Radhika K., Sundaram K.K. (2018). Anatomical Study of Left Coronary Artery and its Variations–Cadaveric Study. J. Clin. Diagn. Res..

[16] Sangita M., Yadav J., Chaurasia J.K., Arora A., Jahan A., Patnaik M. (2023). Hypoplastic coronary artery disease, as a cause of sudden death. Autops. Case Rep..

[17] Liu Y., Lu X., Xiang F.L., Poelmann R.E., Gittenberger-de Groot A.C., Robbins J., Feng Q. (2012). Nitric oxide synthase-3 deficiency results in hypoplastic coronary arteries and postnatal myocardial infarction. Eur. Heart J..

[18] Riede F.N., Bulla S., Grundmann S., Werner M., Riede U.N., Otto C. (2013). Isolated hypoplastic circumflex coronary artery: A rare cause of haemorrhagic myocardial infarction in a young athlete. Diagn. Pathol..

[19] Moazzen H., Lu X., Liu M., Feng Q. (2015). Pregestational Diabetes Induces Fetal Coronary Artery Malformation via Reactive Oxygen Species Signaling. Diabetes.

[20] Greco E.R., Engineer A., Saiyin T., Lu X., Zhang M., Jones D.L., Feng Q. (2022). Maternal nicotine exposure induces congenital heart defects in the offspring of mice. J. Cell. Mol. Med..

[21] Angelini P., Villason S., Chan A., Diez G., Angelini P. (1999). Normal and Anomalous Coronary Arteries in Humans. Coronary Artery Anomalies: A Comprehensive Approach.

[22] Maron B.J., Shirani J., Poliac L.C., Mathenge R., Roberts W.C., Mueller F.O. (1996). Sudden death in young competitive athletes. Clinical, demographic, and pathological profiles. JAMA.

[23] Levin Mtsac R., Degrange Mtsac M., Leacche M., Balaguer J., Byrne J. (2012). Acute Coronary Syndrome Secondary to Hypoplastic Left Main and Left Descending Coronary Arteries. Rev. Argent. Cardiol..

[24] McFarland C., Swamy R., Shah A.P. (2011). Hypoplastic coronary artery disease: A rare cause of sudden cardiac death and its treatment with an implantable defibrillator. J. Cardiol. Cases.

[25] Rodgers J.L., Jones J., Bolleddu S.I., Vanthenapalli S., Rodgers L.E., Shah K., Karia K., Panguluri S.K. (2019). Cardiovascular Risks Associated with Gender and Aging. J. Cardiovasc. Dev. Dis..

[26] Aricatt D.P., Prabhu A., Avadhani R., Subramanyam K., Manzil A.S., Ezhilan J., Das R. (2023). A study of coronary dominance and its clinical significance. Folia Morphol..

[27] Knaapen M., Koch A.H., Koch C., Koch K.T., Li X., Van Rooij P.C., Tijssen J.G., Peters R.J., van der Wal A.C., Damman P. (2013). Prevalence of left and balanced coronary arterial dominance decreases with increasing age of patients at autopsy. A postmortem coronary angiograms study. Cardiovasc. Pathol..

[28] Doi Y., Waki K., Ogino K., Hayashi T. (2021). Hypoplastic coronary artery disease and hypertension in a child: A case report. Eur. Heart J.-Case Rep..

[29] Cubrilo-Turek M. (2003). Hypertension and Coronary Heart Disease. EJIFCC.

[30] Haffner S.M. (1999). Diabetes, hyperlipidemia, and coronary artery disease. Am. J. Cardiol..

[31] Shah P.K., Helfant R.H. (1988). Smoking and Coronary Artery Disease. Chest.

[32] Sim D.S., Jeong M.H., Choi S., Yoon N.S., Yoon H.J., Moon J.Y., Hong Y.J., Kim K.H., Park H.W., Kim J.H. (2009). Myocardial Infarction in a Young Man due to a Hypoplastic Coronary Artery. KCJ.

